# Multi-Omics Analysis of the Anti-tumor Synergistic Mechanism and Potential Application of Immune Checkpoint Blockade Combined With Lenvatinib

**DOI:** 10.3389/fcell.2021.730240

**Published:** 2021-09-09

**Authors:** Yuting Lu, Jiangtao Jin, Qi Du, Min Hu, Yuhan Wei, Miao Wang, Hongzhong Li, Qin Li

**Affiliations:** ^1^Department of Oncology, Beijing Friendship Hospital, Capital Medical University, Beijing, China; ^2^Department of Intervention Therapy, Zezhou People’s Hospital, Jincheng, China; ^3^Department of Biochemistry and Molecular Biology, School of Basic Medical Sciences, Shanxi Medical University, Taiyuan, China; ^4^Chongqing Key Laboratory of Molecular Oncology and Epigenetics, The First Affiliated Hospital of Chongqing Medical University, Chongqing, China

**Keywords:** immune-checkpoint blockade, lenvatinib, synergistic mechanism, multi-omics analysis, malignancy

## Abstract

The combination of immune-checkpoint blockade (ICB) and lenvatinib has demonstrated robust clinical effects that are superior to those of monotherapies, but the synergistic anti-tumor mechanisms remain unclear. Exploring the synergistic molecular mechanisms and early identifying potential application have key importance for clinical therapeutics. We firstly systematically reviewed published data of ICB in combination with lenvatinib for the treatment of cancer by meta-analysis. A subsequent bioinformatics analysis explored the mechanism of combined ICB and lenvatinib therapy in 33 cancer types. Transcriptomic analysis was conducted by RNA-seq, and genomic analysis was performed on gene mutations and copy-number alteration data. Tumor-related pathways and tumor immune micro-environment (TIME) were also investigated. The meta-analysis showed a 38.0% objective response rate (ORR) and 79% disease control rate (DCR) for ICB combined with lenvatinib. Multi-omics analysis revealed that ICB and lenvatinib target genes were highly expressed and showed driving alterations in six specific malignancies. Pathway-enrichment analysis found target genes were implicated in tumor development, angiogenesis, and immunoregulatory associated pathways. This study verified the potential synergistic mechanisms of ICB combined with lenvatinib at transcriptomics, genomics, protein, and cellular levels and recognized nine tumor types had ≥ 2 positive treatment-related molecular characteristics, which might benefit particularly from this combined strategy. The findings would help to provide clinical insights and theoretical basis for optimizing of targeted therapy-immunotherapy combinations, and for guiding individualized precision-medicine approaches for cancer treatment.

## Introduction

Immune-checkpoint blockade (ICB) combined with molecular targeted therapy is an emerging trend in anticancer treatment. The blockade of inhibitory immune checkpoints, including programmed cell death-1 (PD-1) and its ligand programmed cell death ligand-1 (PD-L1), lymphocyte activation gene-3 (LAG-3), and cytotoxic T-lymphocyte–associated antigen 4 (CTLA-4), can reactivate the body’s immune system, thereby preventing the escape of tumor cells (Wei et al., 2018; He and Xu, 2020). In contrast, molecular targeted therapy reverses the malignant biological behaviors of tumors by blocking the tumorigenic components of cells at the molecular level (Druker, 2003). At present, it has been clinically found that the combined application of ICB and anti-angiogenic drugs or targeted therapy have synergistic effects in malignancy, but the molecular mechanism of this combination remains unclear.

The representative ICB therapy, pembrolizumab, is a PD-1 inhibitor, which has been approved for the treatment of colorectal cancer (Powles et al., 2020), non-small cell lung cancer (NSCLC) (Mok et al., 2019) (Abbreviations and full names of tumors and targets was shown in Supplementary Table 1) and other cancer types. Lenvatinib is a novel molecular targeted therapeutic drug that can inhibit a variety of tyrosine kinases, including vascular endothelial growth factor receptor (VEGFRs) 1–3, fibroblast growth factor receptor (FGFRs) 1–4, platelet derived growth factor receptor (PDGFR), stem cell factor receptor (c-kit) and rearranged during transfection (RET) proto-oncogene (Suyama and Iwase, 2018; Al-Salama et al., 2019). It exhibits potent antitumor, antiangiogenic, and immunomodulatory effects. Lenvatinib has been approved for unresectable liver hepatocellular cancer (LIHC) (Kudo et al., 2018), thyroid cancer (Schlumberger et al., 2015), and advanced renal cell carcinoma (RCC) (Motzer et al., 2015).

Nonetheless, the low remission rates with ICB monotherapies and the development of drug resistance with molecular targeted therapies have urged researchers to explore the potential of combination therapies. Data from previous studies showed that the median overall survival (mOS) of patients with advanced LIHC was 12.9 months with pembrolizumab monotherapy (Zhu et al., 2018) and 13.6 months with lenvatinib monotherapy (Kudo et al., 2018), whereas the mOS of patients treated with pembrolizumab and lenvatinib combination therapy reached 22.0 months (Finn et al., 2020). However, the therapeutic effect of ICB combined with lenvatinib is still poorly understood in some cancer types, and few investigations have addressed the mechanisms underlying the synergy of this combination.

Therefore, in this study, we first conducted a meta-analysis to analyze the efficacy of ICB and lenvatinib combination therapy. Bioinformatics analyses were subsequently performed to comprehensively analyze the synergistic mechanisms of ICB combined with lenvatinib and potential efficacies at the transcriptome (RNA-seq), genome (gene mutations, copy-number alterations [CNA]), protein, and immune cell levels.

## Materials and Methods

### Search Strategy and Selection Criteria for the Meta-Analysis

The PubMed, Embase, and Cochrane library databases were systematically queried by two authors of this study (Yuting Lu and Min Hu) for articles pertaining to ICB and lenvatinib. Abstracts from meetings of the European Society for Medical Oncology (ESMO) and the American Society of Clinical Oncology (ASCO) on the same topics were also searched. The latest data were used when the same trial was reported in multiple articles. The data-entry cutoff date was March 1, 2021. The search terms were: “lenvatinib” AND “immunotherapy OR immune therapy OR immune checkpoint inhibitor OR immune checkpoint blockade OR immune vaccine OR nivolumab OR ipilimumab OR pembrolizumab OR atezolizumab OR tremelimumab OR avelumab OR durvalumab” AND “combination OR combined with OR plus” AND “tumor OR cancer OR carcinoma OR neoplasm OR malignancy OR sarcoma.”

The inclusion criteria were as follows: (1) study design: prospective trial; (2) population: patients with pathologically confirmed advanced cancer; (3) intervention: lenvatinib + ICB; (4) study endpoints: objective-response rate (ORR), disease-control rate (DCR). The following information was independently extracted from the articles or abstracts identified by both authors (Yuting Lu and Min Hu): the authors’ names, the journal, the publication year, the tumor type, the treatment regimen, the line of treatment, the sample size per group, and the study endpoints. All information was crosschecked. All disagreements were resolved by discussion with a third reviewer (Qi Du).

### Bioinformatics Data Mining

#### Expression and Correlation Analysis of ICB and Lenvatinib Targets

The target gene expression profile date of tumor samples and normal tissues was obtained from the cancer genome atlas (TCGA) in the UALCAN database^1^. The String database^2^ was employed to construct protein--protein-interaction (PPI) networks. The threshold was set to 0.4 to examine the relationships between the target proteins of ICB and lenvatinib. The UCSC Xena database^3^ was used to determine the Pearson correlation coefficient (*r*) values between the ICB and lenvatinib target genes in pan-cancer analysis. The GEPIA database^4^ was used to obtain the Pearson correlation coefficient of specific cancer types. Visualization was performed by the corrplot package of R (version 4.0.2).

#### Analysis of Genetic Alterations in ICB and Lenvatinib Target Genes

A total of 10967 samples from 32 studies from the TCGA in the cBioPortal database^5^ were used to analyze mutations and CNAs of ICB and lenvatinib target genes in different cancers. To identify likely functional variants, we used the driver-annotation resources OncoKB^6^ and Cancer Hotspots^7^. Passenger mutations, also known as variants of unknown significance, were filtered. The ggplot 2 and pheatmap packages of R (version 4.0.2) were used for visualization.

#### Gene Ontology (GO) and Kyoto Encyclopedia of Genes and Genomes (KEGG) Pathway Analyses of ICB and Lenvatinib Target Genes

To delineate the genetic and metabolic pathways of the ICB and lenvatinib target genes that affect tumor development, progression, and the immune microenvironment, the fifteen target genes were studied in terms of KEGG signaling pathways and GO pathway-enrichment analysis using the R packages clusterProfiler, org.Hs.eg.db, ggplot 2, enrichplot, and GOplot. *P* < 0.05 was set as the cut-off criterion. The enrichment analysis website Reactome^8^ was also used for validation.

#### Correlation Analysis Between ICB and Lenvatinib Target Genes and Tumor-Infiltrating Immune Cells

The TIMER database^9^ was used to obtain correlation coefficients between ICB and lenvatinib target genes, tumor infiltrates immune cells, and the associated pan-cancer survival data in TCGA. Datasets were selected from CIBERSORT (Myeloid-derived suppressor cells [MDSC] data were only found in the TIDE dataset). The pheatmap package of R (version 4.0.2) was used for visualization. The workflow is shown in Supplementary Figure 1.

#### Statistical Analysis

Statistical analysis was performed with Stata SE 16 (Stata Corporation, College Station, TX, United States) and R (version 4.0.2). We calculated the pooled ratio and 95% CI for ORR and DCR. I-squared (*I*^2^) tests were performed to evaluate heterogeneities between trials. In cases where an *I*^2^ of >50% was observed, which indicated the existence of heterogeneity, the randomized-effects model was used. Otherwise, the fixed effect model was adopted. Pearson correlation-coefficient analysis was performed to examine the relationship between ICB and lenvatinib target genes, and that between their targets and immune infiltrates. Kaplan–Meier analysis with a log-rank test was performed for immune infiltrates and genes to evaluate survival differences. The influences of gene-expression levels on survival were evaluated using the Cox proportional-hazards model. *P* < 0.05 was considered to reflect statistically significant differences.

## Results

### Meta-Analysis of Efficacy Data

**FIGURE 1:**
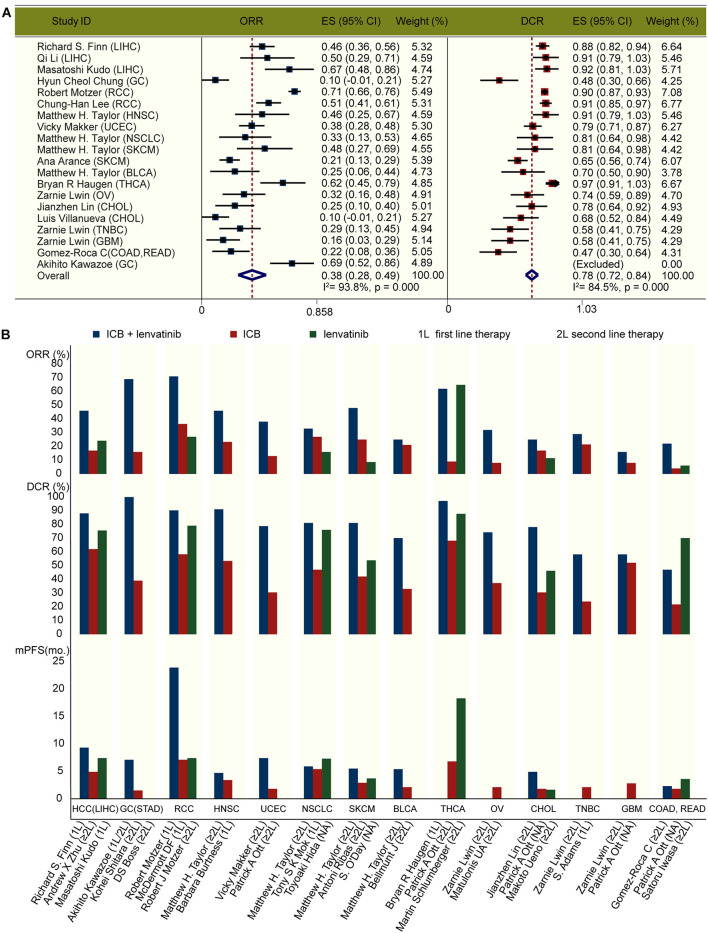
Clinical outcomes with ICB and lenvatinib. **(A)** Forest plot analysis of ORR and DCR with ICB combined with lenvatinib across different cancer types. **(B)** ORR, DCR, and mPFS with pembrolizumab and lenvatinib combined or as monotherapy across different cancer types. 1L, first line therapy; 2L, second line therapy; BLCA, bladder urothelial carcinoma; CHOL, cholangiocarcinoma; COAD, colon adenocarcinoma; ES, effect size; GBM, glioblastoma multiforme; GC, gastric cancer; HNSC, head and neck squamous cell carcinoma; LIHC, liver hepatocellular carcinoma; mo., month; NSCLC, non-small-cell lung cancer; OV, ovarian serous cystadenocarcinoma; RCC, renal cell carcinoma; READ, rectum adenocarcinoma; SKCM, skin cutaneous melanoma; THCA, thyroid carcinoma; TNBC, triple-negative breast carcinoma; UCEC, uterine corpus endometrial carcinoma.

We identified 412 original articles and 56 meeting abstracts during our electronic searches. After screening, four original articles and 12 conference abstracts met the criteria for inclusion in the meta-analysis (Supplementary Figure 2; Fernandez et al., 2020; Finn et al., 2020; Haugen et al., 2020; Kawazoe et al., 2020; Kudo et al., 2020; Lee et al., 2020; Li et al., 2020; Lin et al., 2020; Lwin et al., 2020; Makker et al., 2020; Taylor et al., 2020; Chung, 2021; Gomez-Roca et al., 2021; Motzer et al., 2021; Villanueva, 2021). The key characteristics of the 15 included studies are presented in Table 1. Across these studies, ICB combined with lenvatinib was associated with a benefit in ORR (pooled ratio, 0.38; 95% CI, 0.28–0.49; *P* < 0.001) and DCR (pooled ratio, 0.78; 95% CI, 0.72–0.84; *P* < 0.001) (Figure 1A). The results of the major endpoints with combined ICB and lenvatinib compared with ICB and lenvatinib monotherapy across different cancer types are summarized in Figure 1B (Boss et al., 2012; O’Day et al., 2013; Ribas et al., 2015; Bellmunt et al., 2017; Kudo et al., 2018; Adams et al., 2019; Burtness et al., 2019; Hida et al., 2019; Makker et al., 2019; Matulonis et al., 2019; Mok et al., 2019; Ott et al., 2019; Finn et al., 2020; Haugen et al., 2020; Iwasa et al., 2020; Kawazoe et al., 2020; Lin et al., 2020; Lwin et al., 2020; Powles et al., 2020; Gomez-Roca et al., 2021).

**TABLE 1:** Main characteristics of the studies included in the meta-analysis.

Author	Year	Tumor	Phase	Line	INT	*N*	ORR	DCR	mPFS
	
							(%)	(%)	(mo.)
Robert Motzer	2021	Advanced RCC	III	1L	L + K	355	71.0	90.2	23.9
Hyun Cheol Chung	2021	Advanced GC	II	3L	L + K	31	10.0	48.0	2.5
Luis Villanueva	2021	Advanced CHOL	II	2L	L + K	31	10.0	68.0	6.1
Carlos Gomez-Roca	2021	Advanced colorectal	II	3L	L + K	32	22.0	47.0	2.3
Chung-Han Lee	2020	Metastatic ccRCC	II	≥2L	L + K	104	51.0	91.0	11.7
Akihito Kawazoe	2020	Advanced GC	II	1/2L	L + K	29	69.0	100.0	7.1
Bryan R Haugen	2020	RR-DTC	II	≥2L	L + K	30	62.0	97.0	NE
Vicky Makker	2020	Advanced UCEC	Ib/II	≥2L	L + K	94	38.3	78.7	7.4
Matthew H. Taylor	2020	Advanced SKCM	Ib/II	≥2L	L + K	21	48.0	81.0	5.5
Ana Arance	2020	Advanced SKCM	Ib/II	≥2L	L + K	103	21.4	65.0	4.2
Richard S. Finn	2020	Unresectable HCC	Ib	1L	L + K	104	46.0	88.0	9.3
Qi Li	2020	Unresectable HCC	NE	1L	L + PD-1 inhibitors	22	50.0	90.9	12.1
Masatoshi Kudo	2020	Unresectable HCC	Ib	1L	L + O	24	66.7	91.7	7.4
Matthew H. Taylor	2020	Advanced HNSC	Ib/II	≥2L	L + K	22	46.0	91.0	4.7
Matthew H. Taylor	2020	Advanced NSCLC	Ib/II	≥2L	L + K	21	33.0	81.0	5.9
Zarnie Lwin	2020	Advanced ovarian	II	4L	L + K	31	32.3	74.2	NE
Zarnie Lwin	2020	Advanced TNBC	II	2/3L	L + K	31	29.0	58.1	NE
Jianzhen Lin	2020	Refractory CHOL	NE	≥2L	L + K	32	25.0	78.1	4.9
Matthew H. Taylor	2020	Advanced BLCA	Ib/II	≥2L	L + K	20	25.0	70.0	5.4
Zarnie Lwin	2020	Advanced GBM	II	2L	L + K	31	16.1	58.1	NE

*INT, intervention; N, patient population numbers; ORR, objective response rate; DCR, disease control rate; mPFS, median progression free survival; PD-1, programmed cell death 1; L, lenvatinib; K, pembrolizumab; O, nivolumab; BLCA, bladder urothelial carcinoma; CHOL, cholangiocarcinoma; COAD, colon adenocarcinoma; ccRCC, clear cell renal cell carcinoma; GC, gastric cancer; HNSC, head and neck squamous cell carcinoma; GBM, glioblastoma multiforme; HCC, hepatocellular carcinoma, NSCLC, non-small-cell lung cancer; RR-DTC, radioiodine-refractory differentiated thyroid carcinoma; RCC, renal cell carcinoma; SKCM, skin cutaneous melanoma; TNBC, triple-negative breast carcinoma; UCEC, uterine corpus endometrial carcinoma.*

### Expression Analysis of ICB and Lenvatinib Targets

**FIGURE 2:**
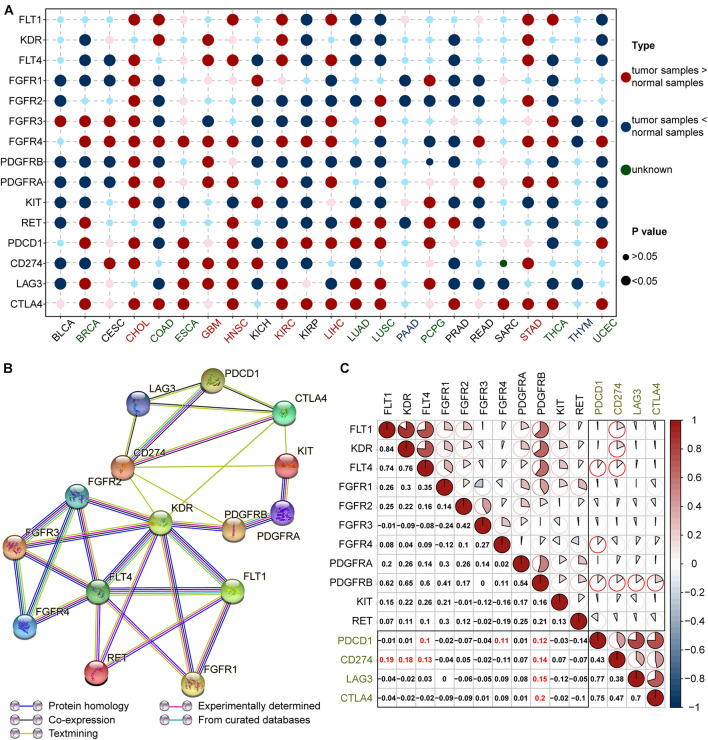
Expression and correlation of ICB and lenvatinib targets. **(A)** Comparison of ICB and lenvatinib target genes expression in tumor tissue and corresponding normal tissue. The cancer types in red text represent high expression of both lenvatinib (≥4 large red circles) and ICB (≥2 large red circles) target genes; green text represents moderate expression defined as higher expression of either lenvatinib (≥4 large red circles) or ICB (≥2 large red circles) target genes; blue text represents low expression of both lenvatinib and ICB target genes (no large red circles for either). **(B)** PPI network construction of lenvatinib and ICB target genes. **(C)** Correlation coefficient graph of ICB and lenvatinib target genes in pan-cancer. Numbers and the pie chart size correspond to the Pearson correlation coefficient (*r* value). The black boxes are the intersecting sections, and the red highlighted text and circles are *r* ≥ 0.1. BRCA, breast invasive carcinoma; CESC, cervical squamous cell carcinoma and endocervical adenocarcinoma; ESCA, esophageal carcinoma; KICH, kidney chromophobe; KIRC, kidney renal clear cell carcinoma; KIRP, kidney renal papillary cell carcinoma; LUAD, lung adenocarcinoma; LUSC, lung squamous cell carcinoma; PAAD, pancreatic adenocarcinoma; PCPG, pheochromocytoma and paraganglioma; PAAD, prostate adenocarcinoma; SARC, sarcoma; STAD, stomach adenocarcinoma; THYM, thymoma; ICB, immune-checkpoint blockade; FLT1 (VEGFR1), vascular endothelial growth factor receptor 1; KDR (VEGFR2), vascular endothelial growth factor receptor 2; FLT4 (VEGFR3), vascular endothelial growth factor receptor 3; FGFR, fibroblast growth factor receptor; PDGFR, platelet derived growth factor receptor; KIT, stem cell factor receptor; RET, the rearranged during transfection; PDCD1 (PD-1), programmed cell death 1; CD274 (PD-L1), programmed cell death ligand 1; LAG3, lymphocyte-activation protein 3; CTLA4, cytotoxic T-lymphocyte-associated antigen 4.

The bubble chart in Figure 2A shows that ICB and lenvatinib target genes are expressed in a broad range of cancers. In cholangiocarcinoma (CHOL), glioblastoma multiforme (GBM), head and neck squamous cell carcinoma (HNSC), kidney clear cell renal cell carcinoma (KIRC), LIHC, and stomach adenocarcinoma (STAD), both ICB and lenvatinib target genes were highly expressed (≥4 red bubbles for lenvatinib and ≥2 red bubbles for ICB, *P* < 0.05) (the red text in the abscissa). This suggests that the application of ICB combined with lenvatinib might be more effective in these malignancies. In contrast, in pancreatic adenocarcinoma (PAAD) and thymoma (THYM), the target genes of both therapies showed low expression (the blue text in the abscissa), which was suggested that this combination might be less effective in these tumors. The expression of ICB and lenvatinib target genes was inconsistent in breast invasive carcinoma (BRCA), colon adenocarcinoma (COAD), esophageal carcinoma (ESCA), lung adenocarcinoma (LUAD), lung squamous cell carcinoma (LUSC), pheochromocytoma and paraganglioma, thyroid carcinoma (THCA), and uterine corpus endometrial carcinoma (UCEC), with target genes for only one of the therapies showing higher expression (≥4 red bubbles for lenvatinib or ≥2 red bubbles for ICB, *P* < 0.05) (the green text in the abscissa). The combination of the two therapies may play a complementary or antagonistic role in these cancer types.

### Correlation Analysis of ICB and Lenvatinib Targets

Protein–protein-interaction network analysis (Figure 2B) showed that the target proteins of ICB and lenvatinib displayed complex and extensive interactions, both directly and indirectly. Specifically, co-expression evidence was observed for the following proteins: VEGFR1, VEGFR2 and VEGFR3; FGFR2, FGFR3, and FGFR4; PD-1, PD-L1, LAG3, and CTLA4; and KIT and PDGFRA (Figure 2B, black lines). Analysis of scientific papers suggested that protein correlations exist between VEGFR2 and PD-L1 or CTLA4, between KIT and PD-L1 or CTLA4, and between PDGFRB and PD-L1 (Figure 2B, green lines). There were also protein correlations derived from experimental results (Figure 2B, purple lines) and curated databases (Figure 2B, blue lines).

Further correlation analysis showed that there was a common co-expression relationship between ICB and lenvatinib target genes in all cancers (Figure 2C). Specifically, strong positive correlations were observed between PDGFRB and CTLA4, LAG3, PDCD1 (PD-1), or CD274 (PD-L1); between FLT4 (VEGFR3) or FGFR4 and PD-1; and between FLT1 (VEGFR1) or KDR (VEGFR2) or VEGFR3 and PD-L1 (*r* ≥ 0.10) (highlighted in red). We calculated the number of positive correlation coefficients *r* ≥ 0.1 between lenvatinib and ICB target genes in specific cancer types. The upper half belonged to the high correlation group, e.g., COAD, READ, GBM, skin melanoma (SKCM) (green title in Supplementary Figure 3). Especially in LIHC, there was a strong positive correlation between PD-1 and FGFR1-4, PDGFRA, PDGFRB, or RET; between PD-L1 and FGFR1, PDGFRA, or PDGFRB; and between LAG3 and FGFR1 (Supplementary Figure 3 and Supplementary Data 1). The combination therapy may therefore have great potential in these tumor types. But, the types of cancers included in clinical trials are limited, and the clinical effects of some cancer types have yet to be confirmed. In contrast, the target genes of both therapies generally exhibited strong negative correlations in THCA, this suggests combination therapy may not be as effective as monotherapy for patients with this cancer. However, due to the complexities of the mechanisms of ICB combined with lenvatinib, the effectiveness of this combination strategy in specific cancer types cannot be fully revealed from the correlation of target genes alone, and further exploration is needed.

### Analysis of Genetic Alterations in ICB and Lenvatinib Targets

**FIGURE 3:**
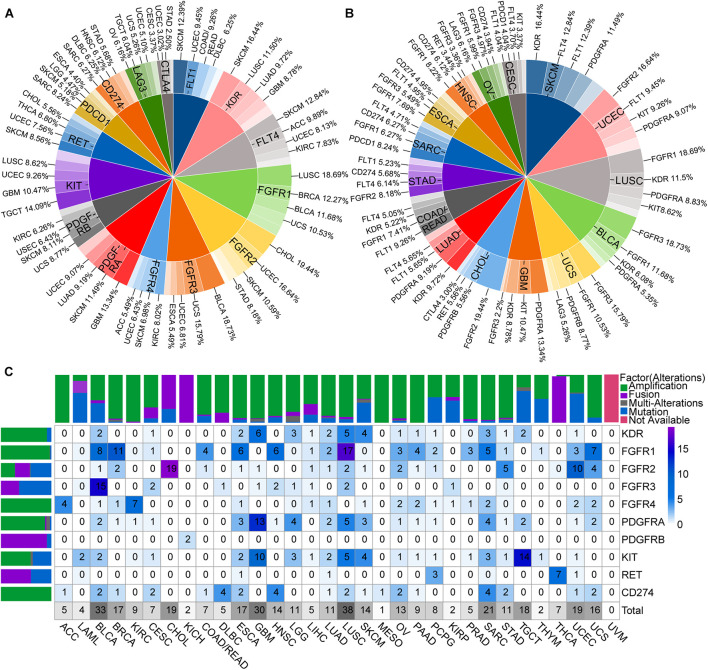
Genomic alterations of ICB and lenvatinib target genes. **(A)** The most common types of cancers with particularly ICB and lenvatinib target genes alterations. **(B)** The most common types of target alterations in each cancer type. **(C)** Driver alterations types and proportions of ICB and lenvatinib target genes in different cancer types. The color of the block represents the proportion of alterations, and the number represents the percentage of alterations. Above is a column chart of alteration types in different cancer types, and on the left is a column chart of alteration types in different targets. ACC, adrenocortical carcinoma; DBLC, diffuse large B-cell lymphoma; LGG, low grade glioma; LAML, acute myeloid leukemia; MESO, mesothelioma; TGCT, testicular germ cell tumors; UCS, uterine carcinosarcoma; UVM, uveal melanoma.

Two-level pie charts were used to summarize the most common types of cancers with particularly ICB and lenvatinib target gene mutations, as well as the most common types of target mutations in each cancer type (Supplementary Data 2 and Figures 3A,B). For example, FGFR4 mutations were common in KIRC and SKCM (Figure 3A), whereas the common target mutations in STAD occurred in FGFR2 and VEGFR3 (Figure 3B). A heat map of the overall mutations in target genes showed that the 15 cancers with the highest mutational loads were SKCM, UCEC, LUSC, bladder urothelial carcinoma (BLCA), LUAD, STAD, uterine carcinosarcoma (UCS), COAD, rectum adenocarcinoma (READ), sarcoma (SARC), ESCA, ovarian serous cystadenocarcinoma (OV), GBM, HNSC, cervical squamous cell carcinoma and endocervical adenocarcinoma (CESC), and CHOL (Supplementary Figure 4 and Supplementary Data 3). This sort order of above-mentioned malignances was closely related to the clinical effect of ICB combined with lenvatinib.

To further explore the driver alterations of target genes in different pathways, mutations and CNA of unknown significance defined by cBioPortal database were excluded as likely passenger alterations. Drive alterations (usually specific hotspot mutations or fusions, amplifications, and multiple alterations involving oncogenes defined by cBioPortal database) are shown in Figure 3C. VEGFR1, VEGFR3, PDCD1, LAG3, and CTLA4 were filtered because of insufficient evidence to drive mutations in cBioPortal database. Therefore, only CD274 (PD-L1) was left as an ICB-target gene. These results demonstrated that driver alterations of ICB and lenvatinib target genes were present in all included cancer types except for uveal melanoma. The cancer types with driver alterations in the lenvatinib target genes occurring at a frequency of ≥9% were as follows (ranked from the highest to the lowest frequency): LUSC, BLCA, GBM, CHOL, testicular germ cell tumors (TGCT), UCEC, SARC, BRCA, ESCA, UCS, SKCM, brain lower grade glioma (LGG), OV, LUAD, KIRC, HNSC, and STAD. The cancer types with a driver alteration in the ICB target gene PD-L1 (amplification in all cases) occurring at a frequency of ≥2% were as follows (ranked from the highest to the lowest frequency): lymphoid neoplasm diffuse large B-cell lymphoma (DLBC), HNSC, SARC, BLCA, CESC, ESCA, LUSC, OV, STAD, and UCS. We also observed that FGFR1 had a high mutation frequency in LUSC and BRCA; FGFR3 had the highest mutation frequency in BLCA. These gene mutations may be the mechanism loci where ICB and lenvatinib play a synergistic role, and explain the clinical efficacy and drug resistance diversity observed for ICB combined with lenvatinib.

### Pathway-Enrichment Analysis of the ICB and Lenvatinib Target Genes

**FIGURE 4:**
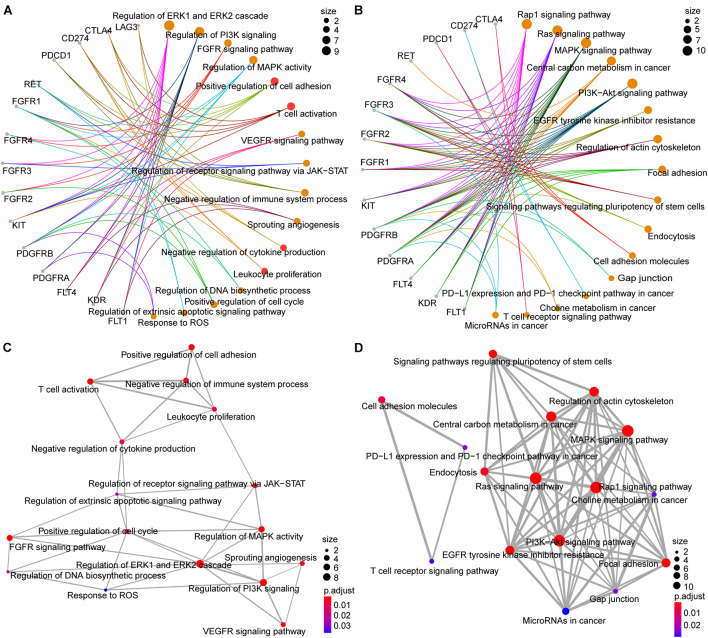
GO and KEGG pathway analysis of lenvatinib and ICB target genes. **(A,B)** Network diagrams between GO terms **(A)** KEGG pathways **(B)** and target genes. **(C,D)** The emapplot of GO terms **(C)** and KEGG pathways **(D)** shown the “pathway–pathway” network. The color of the circle represents the adjusted *P*-value, and the size of the circle represents the number of enriched genes.

The results summarized in Figure 4 show that the ICB and lenvatinib target genes were implicated in multiple pathways associated with tumor development and progression (Supplementary Data 4), including the following terms: Ras signaling pathway, MAPK signaling pathway, PI3K-Akt signaling pathway, regulation of receptor signaling pathway via JAK-STAT, positive regulation of cell cycle. The target genes were also involved in the following antiangiogenic pathways: VEGFR signaling pathway, sprouting angiogenesis, and FGFR signaling pathway. In addition, the target genes of ICB and lenvatinib were significantly enriched in terms of the functional pathways of immune-related genes, including PD-L1 expression and PD-1 checkpoint pathway in cancer, T cell receptor signaling pathway, positive regulation of cell adhesion, T cell activation, and leukocyte proliferation. Finally, the target genes were involved in pathways associated with tumor metabolism, including central carbon metabolism in cancer and choline metabolism in cancer. In addition, this study explored the common pathway connections through which lenvatinib and ICB target genes play a role. KIT, CD274, and CTLA4 are all associated with leukocyte proliferation; KIT, PDCD1, CD274, LAG3, and CTLA4 co-regulatory T cell activation; KDR, RET, PDCD1, CD274, and CTLA4 positive regulation of cell adhesion; FGFR1CD, 274 and LAG3 negative regulation of cytokine production (red circles in Figure 4A). The pathway-network analysis revealed complex interactions among the pathways. Further analysis using the Reactome database (Supplementary Figure 5) showed that the ICB and lenvatinib target genes were significantly enriched in modules such as signal transduction, immune system, disease, hemostasis, metabolism, developmental biology, and extracellular matrix organization. It was further suggested that ICB and lenvatinib-related target genes play roles in multiple pathways related to tumor genesis and development and the immune microenvironment.

### Correlation Analysis Between ICB and Lenvatinib Target Genes, and the Tumor Microenvironment (TME)

**FIGURE 5:**
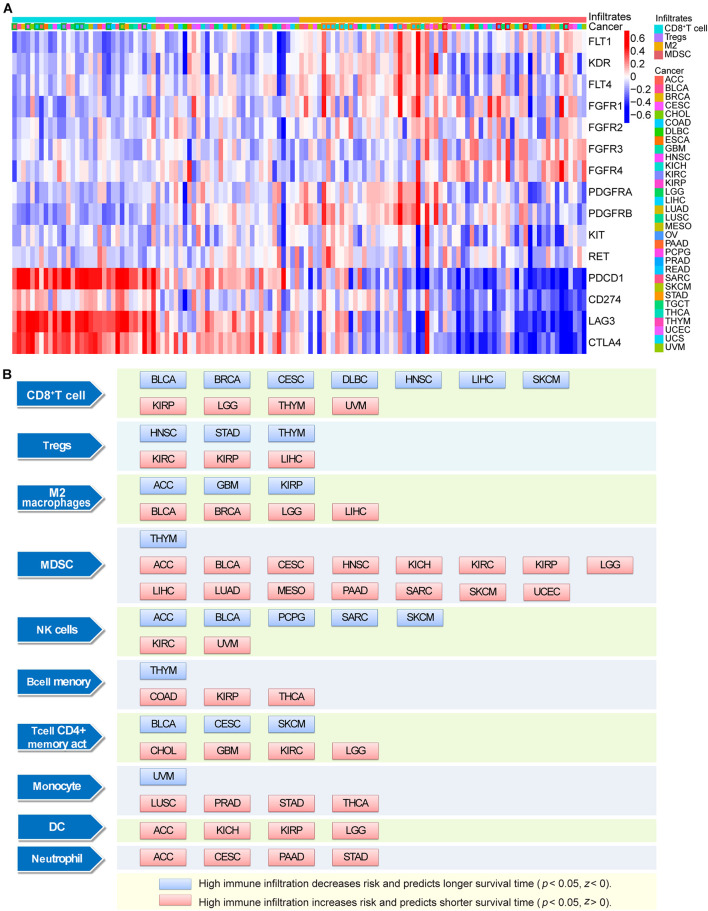
Correlation and survival analysis of immune infiltrating cells with ICB and lenvatinib target genes. **(A)** The correlation between ICB and lenvatinib target genes and tumor infiltration immune cells. The cancer types indicated with a green box if ≥8 positive correlations and more than 4 of them have an *r* ≤ –0.15, and the cancer types indicated with an orange or red box if ≥8 negative correlations and more than 4 of them have an *r* ≥ 0.15. **(B)** The relationship between immune infiltrates and survival outcomes in malignancy. The blue square represents high immune infiltration decreases risk and predicts longer survival time in certain tumors (*P* < 0.05, *Z* < 0), and the red square represents high immune infiltration increases risk and predicts shorter survival time in certain tumors (*P* < 0.05, *Z* > 0). *Z* represents normalized coefficient. DC, dendritic cells; M2, M2 macrophages; MDSC, myeloid-derived suppressor cells.

Analysis using the TIMER database showed that the expression levels of ICB and lenvatinib target genes generally exhibited strong correlations with the levels of immune-cell infiltrates in the TME (Figure 5A and Supplementary Data 5). In most cancers, including adrenocortical carcinoma (ACC), CESC, COAD, DLBC, KIRC, LIHC, LUAD, prostate adenocarcinoma, SKCM and UCEC (the cancer types shown in the green box), the expression levels of lenvatinib target genes generally showed a negative correlation with the levels of CD8^+^ T cell infiltrates (≥8 blue squares and more than four of them have an *r* ≤ –0.15). However, the expression levels of lenvatinib target genes showed positive correlations with the infiltration levels of the immunosuppressive Tregs, M2 macrophages, and MDSCs. Specifically, strong positive correlations (≥8 red squares and more than 4 of them have *r* ≥ 0.15) were observed with levels of M2 macrophages in COAD, DLBC, ESCA, HNSC, KIRC, READ, STAD, TGCT, THYM, and UCS (the cancer types in orange box), and of MDSCs in ACC, kidney renal papillary cell carcinoma (KIRP), LIHC, OV and THCA (the cancer types in red box). ICB and lenvatinib target genes may jointly influence the level of immune cell infiltration through synergistic or antagonistic effects.

Survival analysis (Figure 5B) showed that the presence of CD8 + T cells was associated with a favorable prognosis in BLCA, BRCA, CESC, DLBC, HNSC, LIHC, and SKCM, and the highest infiltration levels were predictive of prolonged survival (blue square). The presence of immune cells was identified as a risk factor for poor prognosis in certain cancer types (red square), e.g., Tregs in KIRC, KIRP, and LIHC; M2 macrophages in BLCA, BRCA, LGG, and LIHC; and MDSCs in ACC, BLCA, CESC, HNSC, kidney chromophobe, KIRC, KIRP, LGG, LIHC, LUAD, mesothelioma, PAAD, SARC, SKCM, and UCEC, and immune cell infiltrates were predictive of shorter survival in these malignancies.

When the bioinformatics results were considered together, we found nine tumor types including LIHC, STAD, KIRC, UCEC, LUAD, SKCM, HNSC, CHOL, and GBM with ≥2 positive treatment-related molecular biological characteristics: high expression of ICB and lenvatinib target genes; high mutational loads and high frequency of driver alterations in lenvatinib and ICB target genes; negatively correlated with CD8^+^ T cell infiltration in lenvatinib target genes (Supplementary Table 2).

## Discussion

Lenvatinib is a multitargeting kinase inhibitor that effectively inhibits the activities of a broad spectrum of targets (Supplementary Table 3). It also exhibits unique characteristics in terms of its IC_50_ value (Supplementary Table 4; Wang et al., 2004; Tian et al., 2011; Stjepanovic and Capdevila, 2014; Kudo, 2018; Lin et al., 2018; Xie et al., 2018) and type-V target-binding mode (Supplementary Table 5; Wang and Long, 2011; Cong-min et al., 2013; Watanabe Miyano et al., 2016; Mori et al., 2017; Roskoski, 2017; Apsel Winger et al., 2019; Kinoshita, 2019). These properties enable lenvatinib to significantly inhibit tumor growth in many types of malignant cancers. Clinical trials of lenvatinib are currently underway (Supplementary Table 6). ICB combined with antiangiogenic therapy is one of the most promising immunotherapeutic combinations. Findings from the present meta-analysis confirm that PD-L1/PD-1 inhibitors combined with lenvatinib result in high ORR, and DCR across patients with multiple different cancer types. However, the complementary and synergistic mechanisms between the two therapy types are currently poorly understood. In the present study we analyzed this topic in detail from a bioinformatics perspective.

The overexpression of lenvatinib target genes and inhibitory immune-checkpoint genes, the positive correlation between the expression levels of these genes, and extensive interactions among the target proteins have suggested the potential for combination therapy (Patel and Minn, 2018; Li et al., 2021). In our expression analysis, the target genes of both ICB and lenvatinib were highly expressed in CHOL, GBM, HNSC, KIRC, LIHC, SKCM, and STAD. This was consistent with the findings from our meta-analysis, which showed that the combination of ICB and lenvatinib achieved better therapeutic effects than either therapy alone in these cancers (Figure 1B). In PAAD and THYM, the target genes of both therapies were not highly expressed, suggesting that combination therapy might not be effective against these cancers, however, this still needs to be clarified in future clinical studies. Combined with a target gene correlation analysis, we found the lenvatinib target genes VEGFR1, VEGFR3, FGFR3, FGFR4, PDGFRA, and the ICB target gene PD-1 were all highly expressed in LIHC, and a relatively strong positive correlation was observed between PD-1 and FGFR3, FGFR4, or PDGFRA (*r* ≥ 0.15) (Supplementary Figure 3). Accordingly, in clinical trials conducted in unresectable LIHC, the efficacy of combination therapy was superior to that of monotherapy (Figure 1B; Kudo et al., 2018; Zhu et al., 2018; Finn et al., 2020). In THCA, there was an overall strong negative correlation between PD-1/PD-L1 and lenvatinib target genes. Data from different clinical trials in thyroid cancer have shown comparable ORRs with combination therapy (62.0%) and lenvatinib monotherapy (64.8%) (Figure 1B; Schlumberger et al., 2015; Haugen et al., 2020). However, whether lenvatinib monotherapy is inferior to combination therapy in THCA requires further verification in phase III randomized clinical trials. Therefore, it is evident that the expression levels and correlations among lenvatinib and ICB target genes and proteins are closely linked to clinical efficacy. Further studies are required to investigate whether these highly expressed and highly correlated targets can be used as biomarkers for predicting clinical efficacy.

Immune-checkpoint blockade and lenvatinib target genes in tumor-promoting pathways exhibit extensive driver alterations, which may explain the improved efficacy of combination therapy in across multiple cancer types (Le et al., 2015; Bielski et al., 2018). The total mutational loads of the lenvatinib and ICB target genes in pan-cancer are highly consistent with the tumor mutation burden and the degrees of immunogenicity of different types of cancer (Lawrence et al., 2013). The increased production of tumor neoantigens resulting from targeted therapy, together with the high mutational load, further increases the immunogenicity of a tumor. This promotes the extensive infiltration of immune cells and increases immune cell activity, both of which are key factors that enhance the clinical benefit of ICB (Cristescu et al., 2018; Chan et al., 2019). Therefore, cancers with a high number of driver alterations in lenvatinib target genes and a high total mutational load in lenvatinib and ICB target genes may be candidates for combination therapy, e.g., in SKCM, UCEC, LUSC, BLCA, LUAD, STAD, UCS, SARC, ESCA, OV, GBM, HNSC, and CHOL. Interestingly, the total mutational load of LIHC was not high, but lenvatinib targets exhibited extensive driver alterations and were generally highly expressed. Additionally, PD-1 displayed relatively strong positive correlations with FGFR3 and FGFR4 (*r* ≥ 0.15). These findings are consistent with the unsatisfactory therapeutic effects of single-agent immunotherapy and the enhanced efficacy of combination therapy observed in LIHC (Kudo et al., 2018; Zhu et al., 2018; Finn et al., 2020). In addition, PD-L1 and VEGFR2 in GBM, CTLA4 and PDGFRA in SKCM, as well as PD-L1 and FGFR2 in STAD were all highly expressed genes with driver alterations (Supplementary Table 7). These data may help to inform future explorations of dual-target therapeutic strategies or drug development. However, gene-expression levels, the presence of driver alterations, and the mutational load cannot precisely predict the efficacy of combination therapies for all cancers, as this is also affected by other factors, such as vascular anomalies (Tian et al., 2017), the abundance of immune cell infiltrates in the TME (Roelands et al., 2020), and the heterogeneity of tumor metabolism (Bergers and Fendt, 2021).

Pathway-enrichment analysis showed that the ICB and lenvatinib target genes were implicated in multiple pathways associated with tumor development and progression, angiogenesis, immunotherapy, and tumor metabolism. Additionally, complex interactions among the pathways were observed. The lenvatinib target genes were significantly enriched in multiple pathways associated with tumor development and progression, such as the RTK–RAS–MAPK signaling pathway, the PI-3 kinase signaling pathway, the JAK–STAT signaling pathway, the cell cycle pathway, and the FGFR–FGF signaling pathway. Blockade of these target genes should inhibit tumor cell proliferation or induce tumor cell apoptosis. Because the lenvatinib target genes are associated with signal-transduction pathways downstream of the TCR [e.g., the MAPK–MEK pathway (Ebert et al., 2016) and the PI3K-AKT-mTORpathway (Pollizzi and Powell, 2015)], targeted therapy can also modulate T cells and other immune cells. With regarding to pathways related to angiogenesis, the triple inhibition of VEGFR, FGFR, and PDGFR can inhibit angiogenesis and increase vascular permeability, which rebuilds the TME and increases the local infiltration of immune cells (Naoum et al., 2018; Cabanillas et al., 2019; Yi et al., 2019). Furthermore, the inhibition of FGFR1–4 can help overcome resistance to antiangiogenic drugs (Lee and Secord, 2014; Zheng et al., 2016). Among the immune-related pathways, T cell activation was regulated by KIT, PD-1, PD-L1, LAG3, and CTLA4; cell adhesion was upregulated by VEGFR2, RET, PD-1, PD-L1, and CTLA4; and leukocyte proliferation was regulated by KIT, PD-L1, and CTLA4 (red circles in Figure 4A). For tumor metabolism-related pathways, ICB and lenvatinib can inhibit tumor cell proliferation by downregulating glycolytic enzymes and reducing mTOR activity in tumor cells (Chang et al., 2015). It is evident that the antitumor effect of ICB combined with lenvatinib is modulated by several targets across multiple pathways.

**FIGURE 6:**
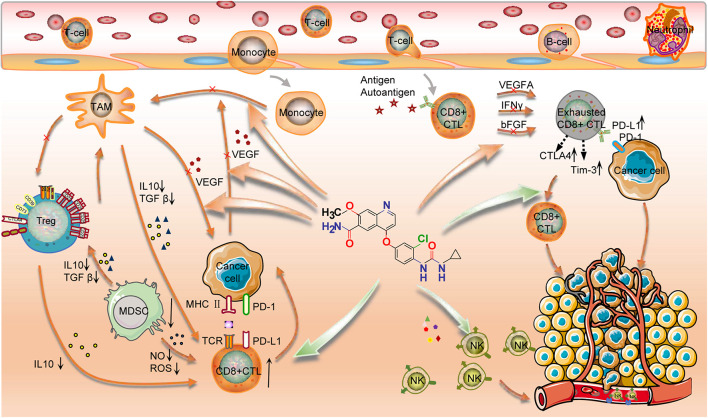
Mechanisms of action of lenvatinib in combination with ICB. Lenvatinib promoted the number and function of CD8^+^ T cells and inhibited the number and function of Tregs and Macrophages M2; Lenvatinib enhanced the cytotoxicity of NK cells; Lenvatinib decreased the expression of PD-1, CTLA-4, and TIM3 in T cells and inhibited the exhaustion of T cells; Lenvatinib also inhibits tumor angiogenesis and abnormalities by inhibiting the secretion of angiogenic factors, such as VEGF, FGF, and PDGF, etc. Finally, PD-1/PD-L1 Ab restores the exhausted T cell activity to kill the cancer cell. Therefore, this combination has a synergistic anti-tumor effect.

In this study, we also explored the impact of ICB and lenvatinib target genes on the tumor immune microenvironment at the immune cell level. In most cancer types, the expression levels of lenvatinib target genes generally showed a negative correlation with the level of CD8^+^ T cell infiltration, suggesting that lenvatinib treatment may increase the degree of infiltration of CD8^+^ T cells. In ACC, KIRP, LIHC, OV, and THCA, the expression levels of lenvatinib target genes generally showed a negative correlation with MDSC infiltration levels, suggesting that lenvatinib treatment may reduce the degree of infiltration of immunosuppressive cells. The combined use of lenvatinib and ICB to promote CD8^+^ T cell infiltration and inhibit the number and function of Tregs, M2 macrophages, or MDSCs (in terms of IL10 or TGF-β secretion) has been confirmed in murine models of LIHC (Kimura et al., 2018; Kudo, 2019), COAD (Kato et al., 2019), and THCA (Gunda et al., 2019). Lenvatinib can also reduce tumor PD-L1 level to improve anti-PD-1 efficacy by blocking FGFR4 (Yi et al., 2021). The mechanisms of action of lenvatinib include (Figure 6): downregulating the expression of PD-1, CTLA-4, and TIM3 in T cells; blocking the binding of VEGFA and bFGF to their receptors, thereby inhibiting T-cell exhaustion (Deng et al., 2020); and destroying tumor cells, leading to the release of tumor antigens, which in turn promotes IFN-γ-mediated antigen presentation (Kato et al., 2019) and enhances the cytotoxicity of natural killer cells (Zhang et al., 2019). Lenvatinib also impedes angiogenesis and vascular anomalies by inhibiting the secretion of angiogenic factors, such as VEGF, FGF, and PDGF, thereby exerting an immunomodulatory effect (Matsuki et al., 2018). In summary, the synergistic antitumor effect of combined lenvatinib and ICB therapy is achieved by reversing the suppression of the body’s immune functions, increasing tumor immunogenicity, promoting tumor vascular normalization, as well as interact at the transcriptome, genome, and protein levels.

There were certain limitations to our study. Firstly, even though our study covered majority of cancer types, number of tumor types were excluded such as most hematologic cancers. Secondly, although our analyses included relatively large sample sets, larger sample sets are required for further exploration.

## Conclusion

This meta-analysis confirmed that ICB combined with lenvatinib exerted greater clinical benefits than monotherapy, across multiple cancers. A multi-omics approach further identified complex and extensive networks connecting the ICB and lenvatinib targets by performing analyses at the gene, protein, and cellular levels, as well as correlation analysis between tumor and immune micro-environment. Most targets were mutually promotional and synergistic, but some of them displayed antagonistic effects. However, the relationships between some targets remained unclear. We found nine tumor types including LIHC, STAD, KIRC, UCEC, LUAD, SKCM, HNSC, CHOL, and GBM with ≥2 positive treatment-related molecular biological characteristics. These findings provide insights for the development of targeted therapy–immunotherapy combinations, and for guiding individualized precision-medicine approaches for cancer treatment.

## Data Availability Statement

The datasets presented in this study can be found in online repositories. The names of the repository/repositories and accession number(s) can be found in the article/Supplementary Material.

## Author Contributions

YL, QL, QD, and JJ conceptualized and designed the manuscript. YL, QD, YW, MW, MH, HL, and QL acquired, analyzed, and interpreted the data. YL wrote and edited the manuscript. YL, JJ, QD, YW, MW, MH, HL, and QL reviewed and revised the manuscript. All authors contributed to the article and approved the submitted version.

## Conflict of Interest

The authors declare that the research was conducted in the absence of any commercial or financial relationships that could be construed as a potential conflict of interest.

## Publisher’s Note

All claims expressed in this article are solely those of the authors and do not necessarily represent those of their affiliated organizations, or those of the publisher, the editors and the reviewers. Any product that may be evaluated in this article, or claim that may be made by its manufacturer, is not guaranteed or endorsed by the publisher.

## Footnotes

1
http://ualcan.path.uab.edu/

2
https://string-db.org

3
https://xenabrowser.net/

4
http://gepia.cancer-pku.cn/detail.php

5
https://www.cbioportal.org/

6
www.oncokb.org

7
www.cancerhotspots.org

8
https://reactome.org/

9
http://timer.cistrome.org/
