# Supplementary material for: Multi-Omics Analysis of the Anti-tumor Synergistic Mechanism and Potential Application of Immune Checkpoint Blockade Combined With Lenvatinib
Source: Front Cell Dev Biol. 2021 Sep 9;9:730240. doi: 10.3389/fcell.2021.730240 (PMC8458708; doi:10.3389/fcell.2021.730240)
Supplement: Supplementary file 1 [file Image_1.PDF]

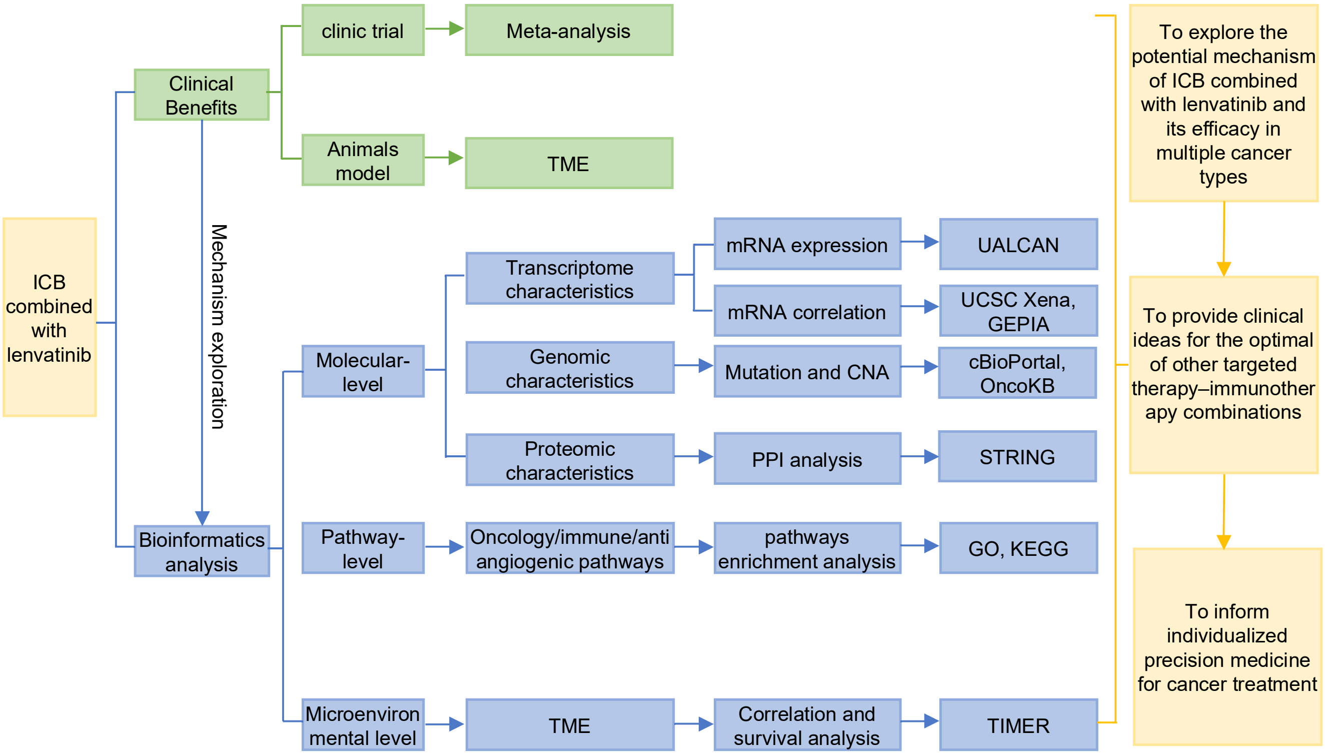

Supplementary Figure 1. Datasets and Workflow. Abbreviations: ICB, immune-checkpoint blockade; TME, tumor microenvironment; CNA, copy-number alterations; GO, Gene ontology; KEGG, Kyoto encyclopedia of genes and genomes.
