# Supplementary figures and images for: Multi-Omics Analysis of the Anti-tumor Synergistic Mechanism and Potential Application of Immune Checkpoint Blockade Combined With Lenvatinib
Source: Front Cell Dev Biol. 2021 Sep 9;9:730240. doi: 10.3389/fcell.2021.730240 (PMC8458708; doi:10.3389/fcell.2021.730240)

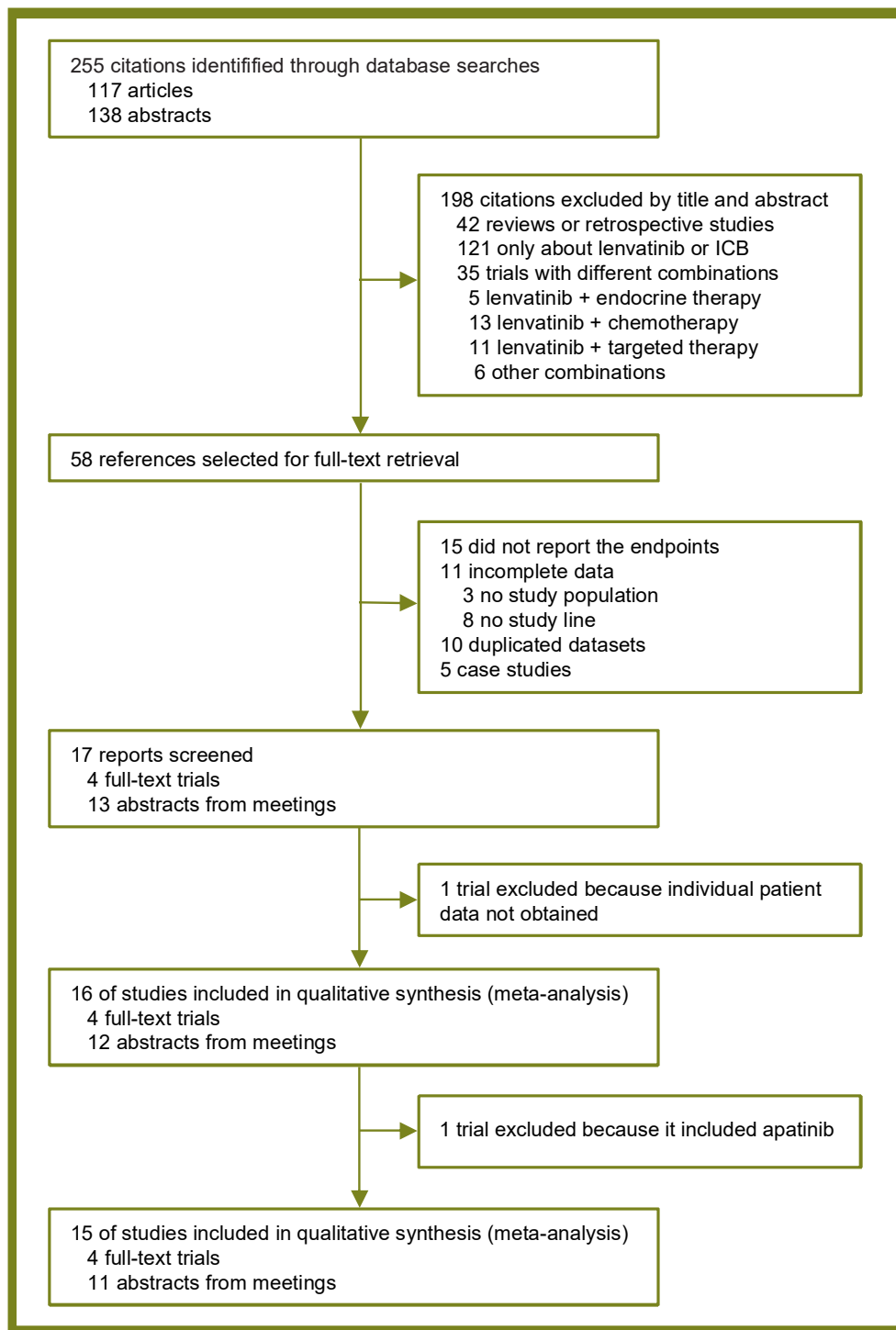

**Supplementary Figure 2. Flow Diagram of the Study Selection Process.**

Supplement: Supplementary file 2 [file Image_2.PDF]
