# Supplementary material for: Multi-Omics Analysis of the Anti-tumor Synergistic Mechanism and Potential Application of Immune Checkpoint Blockade Combined With Lenvatinib
Source: Front Cell Dev Biol. 2021 Sep 9;9:730240. doi: 10.3389/fcell.2021.730240 (PMC8458708; doi:10.3389/fcell.2021.730240)
Supplement: Supplementary file 3 [file Image_3.PDF]

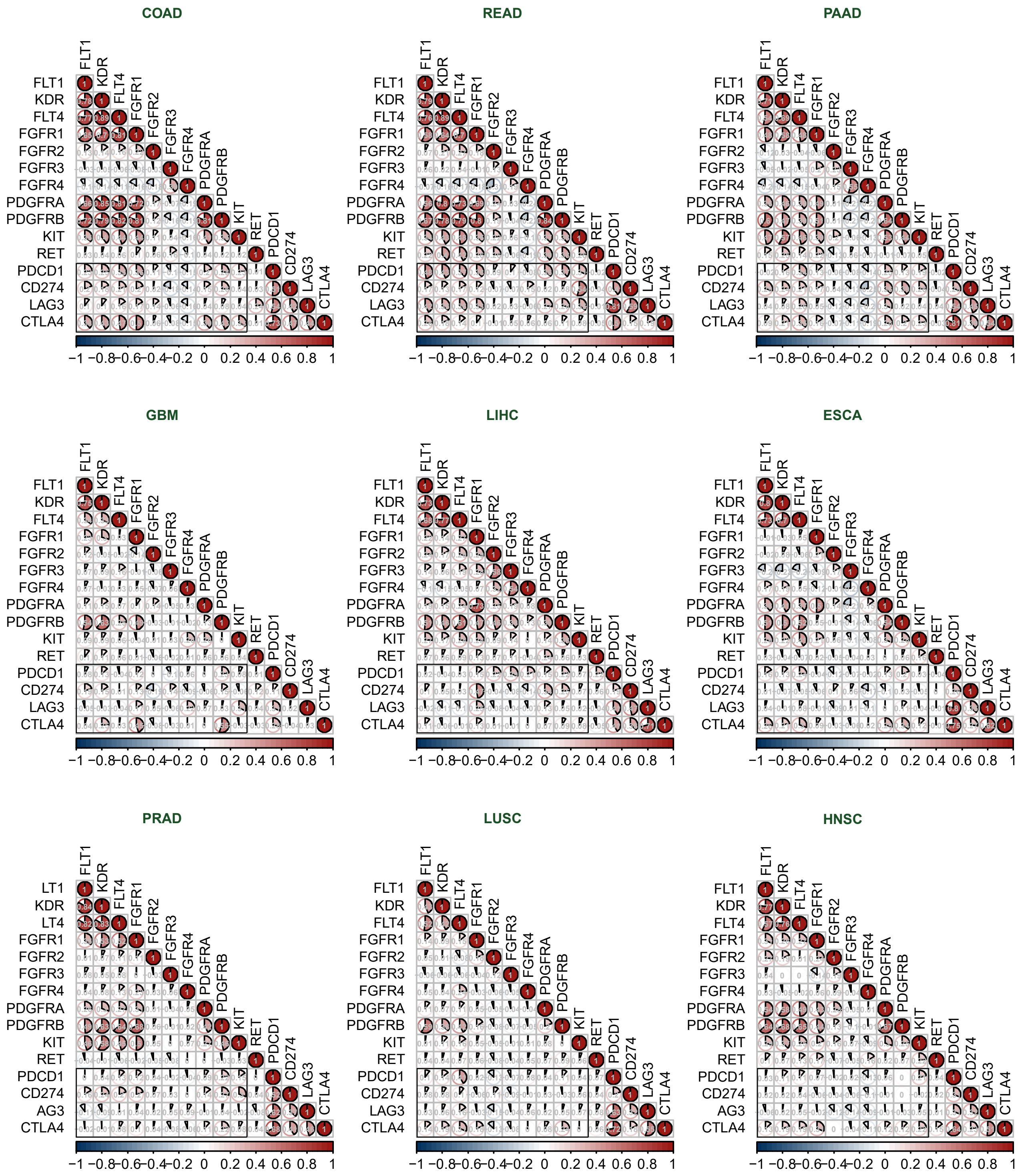
COAD
READ
PAAD
GBM
LIHC
ESCA
PRAD
LUSC
HNSC
FLT1
KDR
FLT4
FGFR1
FGFR2
FGFR3
FGFR4
PDGFRA
PDGFRB
KIT
RET
PDCD1
CD274
LAG3
CTLA4
LT1
LT4
AG3
−1 −0.8 −0.6 −0.4 −0.2 0 0.2 0.4 0.6 0.8 1

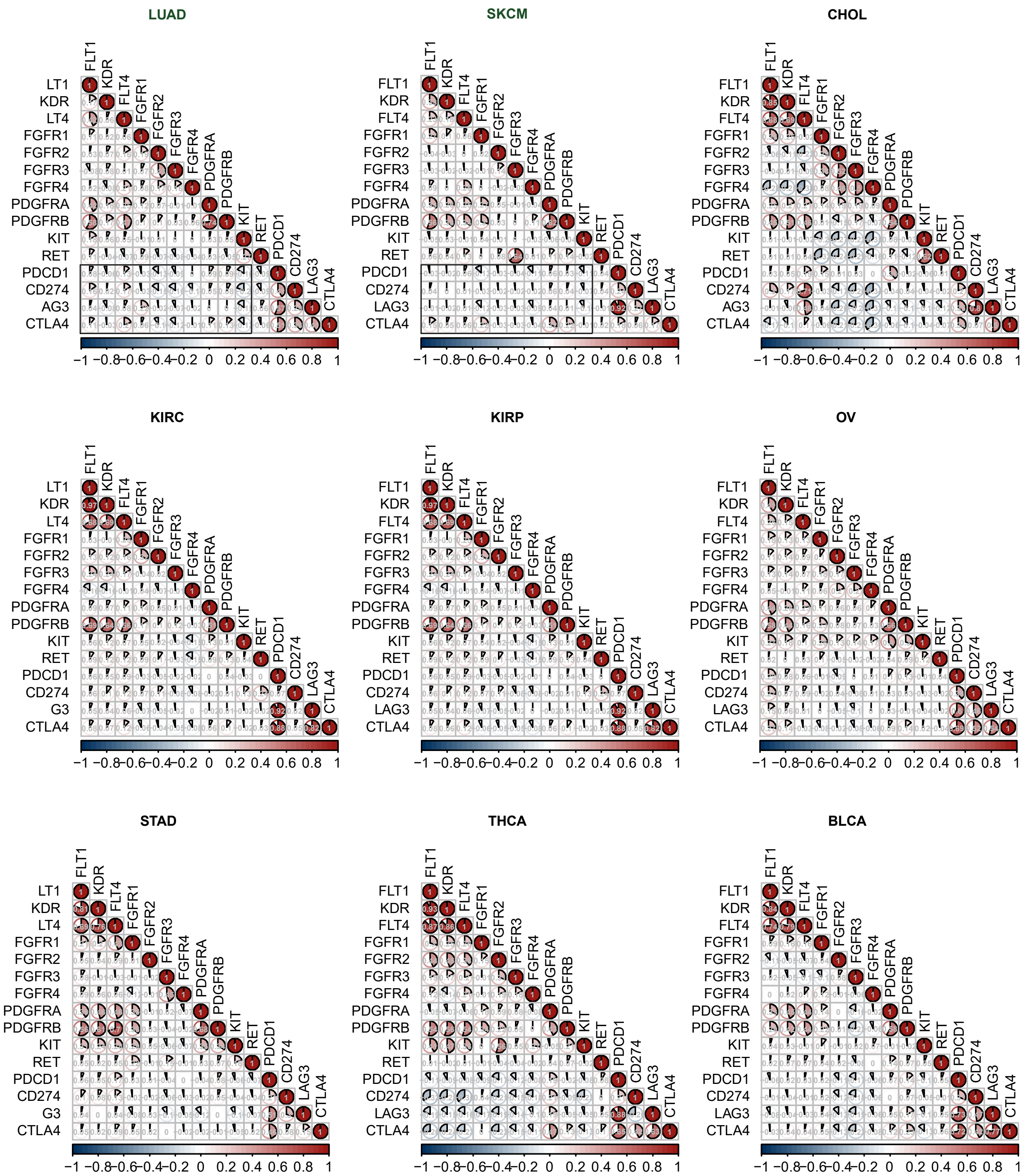
LUAD
SKCM
CHOL
KIRC
KIRP
OV
STAD
THCA
BLCA
FLT1
KDR
FLT4
FGFR1
FGFR2
FGFR3
FGFR4
PDGFRA
PDGFRB
KIT
RET
PDCD1
CD274
LAG3
CTLA4
−1 −0.8 −0.6 −0.4 −0.2 0 0.2 0.4 0.6 0.8 1

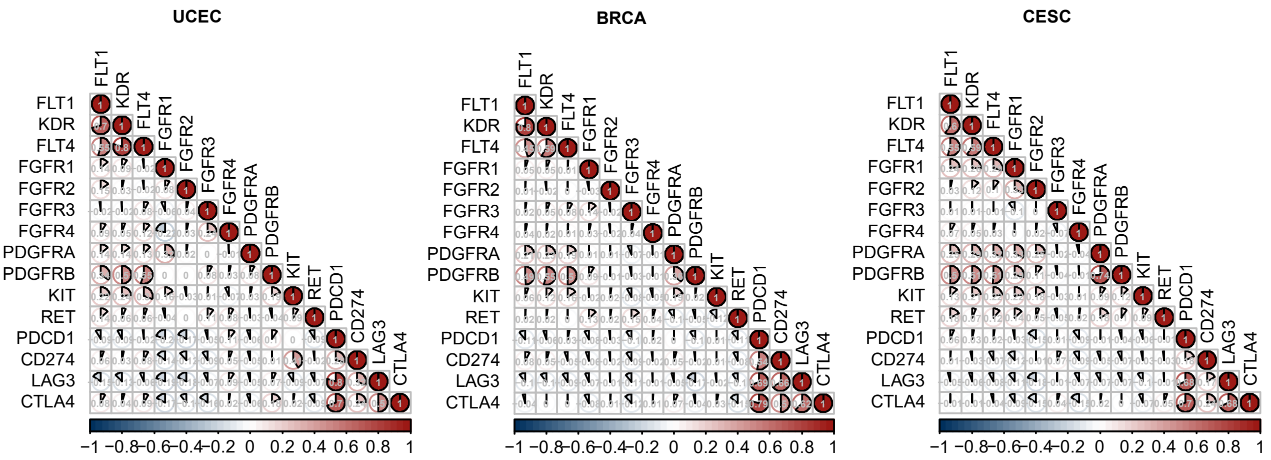

Supplementary Figure 3. Associations between ICB and lenvatinib targets in specific cancer types. Correlation coefficient graph of ICB and lenvatinib target genes in specific cancer types. Numbers correspond to the Pearson correlation coefficient (r value). The upper half of the number of the positive correlation coefficient r ⩾ 0.1 between lenvatinib and ICB target genes belong to the group of high-correlation cancers. The color of the title is in green and the intersecting sections is in black box. The lower half belong to the group of low-correlation cancers, and the color of the title is in black. The pie chart segment represents the r value: larger segments indicate a greater absolute r value; red indicates a positive correlation; blue indicates a negative correlation.

Abbreviations: PPI, protein-protein interaction network; BLCA, bladder urothelial carcinoma; BRCA, breast invasive carcinoma; CHOL, cholangiocarcinoma; COAD, colon adenocarcinoma; ESCA, esophageal carcinoma; GBM, glioblastoma multiforme; HNSC, head and neck squamous cell carcinoma; KIRC, kidney renal clear cell carcinoma; LIHC, liver hepatocellular carcinoma; LUSC, lung squamous cell carcinoma; OV, ovarian serous cystadenocarcinoma; READ, rectum adenocarcinoma; SKCM, skin cutaneous melanoma; STAD, stomach adenocarcinoma; THCA, thyroid carcinoma; UCEC, Uterine corpus endometrial carcinoma.
