# Supplementary material for: Multi-Omics Analysis of the Anti-tumor Synergistic Mechanism and Potential Application of Immune Checkpoint Blockade Combined With Lenvatinib
Source: Front Cell Dev Biol. 2021 Sep 9;9:730240. doi: 10.3389/fcell.2021.730240 (PMC8458708; doi:10.3389/fcell.2021.730240)
Supplement: Supplementary file 4 [file Image_4.PDF]

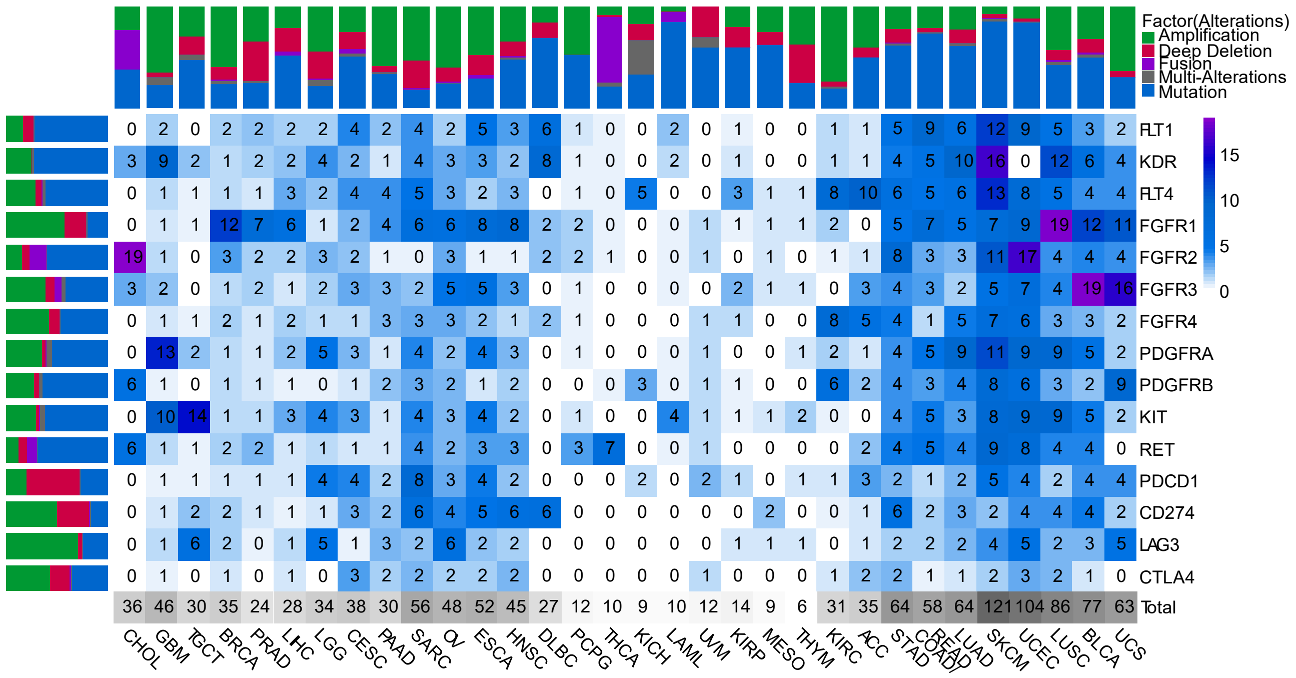

Supplementary Figure 4. ICB and lenvatinib target gene mutation characteristics before filtering for likely passenger mutations. The color of the block represents the proportion of mutations, and the number represents the percentage of mutations. Above is a column chart of mutation types in different cancer types, and on the left is a column chart of mutation types in different targets.

Abbreviations: Abbreviations and full names of tumors and targets are shown in Supplementary Table 1.
