# Supplementary material for: Multi-Omics Analysis of the Anti-tumor Synergistic Mechanism and Potential Application of Immune Checkpoint Blockade Combined With Lenvatinib
Source: Front Cell Dev Biol. 2021 Sep 9;9:730240. doi: 10.3389/fcell.2021.730240 (PMC8458708; doi:10.3389/fcell.2021.730240)
Supplement: Supplementary file 5 [file Image_5.PDF]

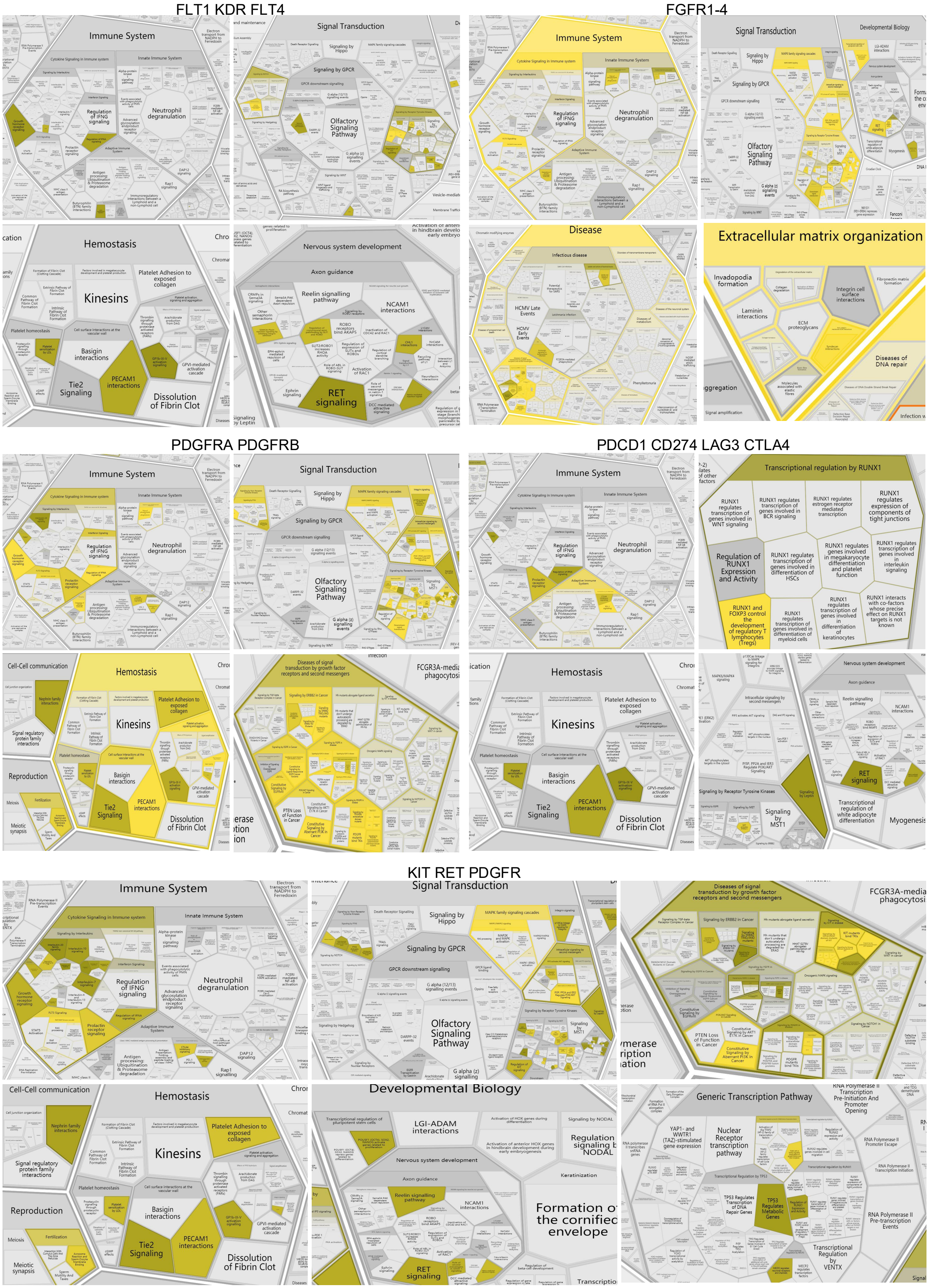
FLT1 KDR FLT4
FGFR1-4
PDGFRA PDGFRB
PDCD1 CD274 LAG3 CTLA4
KIT RET PDGFR
Immune System
Signal Transduction
Hemostasis
Nervous system development
Disease
Extracellular matrix organization
Transcriptional regulation by RUNX1
Developmental Biology
Generic Transcription Pathway
Kinesins
Olfactory Signaling Pathway
RET signaling
Tie2 Signaling
PECAM1 interactions
Dissolution of Fibrin Clot
Basigin interactions
Cell-Cell communication
Reproduction
Diseases of signal transduction by growth factor receptors and second messengers
Formation of the cornified envelope

Supplementary Figure 5. Macro map of pathway enrichment of ICB and lenvatinib target genes. Yellow indicates a significant enrichment module for related targets ($P \leq 0.05$).
