# Supplementary material for: Multi-Omics Analysis of the Anti-tumor Synergistic Mechanism and Potential Application of Immune Checkpoint Blockade Combined With Lenvatinib
Source: Front Cell Dev Biol. 2021 Sep 9;9:730240. doi: 10.3389/fcell.2021.730240 (PMC8458708; doi:10.3389/fcell.2021.730240)
Supplement: Supplementary file 6 [file Table_1.DOCX]

**Supplementary Table 1. Abbreviations-full names of tumors and target genes**

| Abbreviation | Full name |
| --- | --- |
| ACC | Adrenocortical carcinoma |
| BLCA | Bladder urothelial carcinoma |
| BRCA/BC/TNBC | Breast invasive carcinoma/breast carcinoma/Triple-negative breast carcinoma |
| CESC | Cervical squamous cell carcinoma and endocervical adenocarcinoma |
| CHOL | Cholangiocarcinoma |
| COAD | Colon adenocarcinoma |
| DLBC | Lymphoid neoplasm diffuse large B-cell lymphoma |
| ESCA | Esophageal carcinoma |
| GBM | Glioblastoma multiforme |
| HNSC | Head and neck squamous cell carcinoma |
| KICH | Kidney chromophobe |
| KIRC/ccRCC/RCC | Kidney clear cell renal cell carcinoma/Renal cell carcinoma |
| KIRP | Kidney renal papillary cell carcinoma |
| LAML | Acute myeloid leukemia |
| LGG | Brain lower grade glioma |
| LIHC/HCC | Liver hepatocellular carcinoma /Hepatocellular carcinoma |
| LUAD/NSCLC | Lung adenocarcinoma/Non-small-cell lung cancer |
| LUSC/NSCLC | Lung squamous cell carcinoma/Non-small-cell lung cancer |
| MESO | Mesothelioma |
| OV | Ovarian serous cystadenocarcinoma |
| PAAD | Pancreatic adenocarcinoma |
| PCPG | Pheochromocytoma and Paraganglioma |
| PRAD | Prostate adenocarcinoma |
| READ | Rectum adenocarcinoma |
| SARC | Sarcoma |
| SKCM | Skin cutaneous melanoma |
| STAD/GC | Stomach adenocarcinoma/Gastric cancer |
| TGCT | Testicular germ cell tumors |
| THCA/RR-DTC | Thyroid carcinoma/Radioiodine-refractory differentiated thyroid carcinoma |
| THYM | Thymoma |
| UCEC | Uterine corpus endometrial carcinoma |
| UCS | Uterine carcinosarcoma |
| UVM | Uveal melanoma |
| GIST | Gastrointestinal stromal tumor |
| FLT1/FLT4 | VEGFR1/VEGFR3, Vascular endothelial growth factor receptor 1/3 |
| KDR | VEGFR2, Vascular endothelial growth factor receptor 2 |
| FGFR | Fibroblast growth factor receptor |
| PDGFR | Platelet derived growth factor receptor |
| CD274 | Programmed cell death ligand 1 |
| PDCD1 | PD-1, programmed cell death 1 |
| LAG3 | Lymphocyte-activation protein 3 |
| CTLA4 | Cytotoxic T-lymphocyte-associated antigen 4 |
