# Supplementary material for: Multi-Omics Analysis of the Anti-tumor Synergistic Mechanism and Potential Application of Immune Checkpoint Blockade Combined With Lenvatinib
Source: Front Cell Dev Biol. 2021 Sep 9;9:730240. doi: 10.3389/fcell.2021.730240 (PMC8458708; doi:10.3389/fcell.2021.730240)
Supplement: Supplementary file 7 [file Table_2.DOCX]

**Supplementary Table 2. Predicted treatment efficacy of combined immune checkpoint inhibitor and lenvatinib in multiple tumor types**

| Tumor | High ICB and lenvatinib target gene expression | High driver mutation of lenvatinib targets and high total mutation load of ICB and lenvatinib targets | Lenvatinib target genes negatively correlated with CD8+ T cells |
| --- | --- | --- | --- |
| ACC |  |  | + |
| BLCA |  | + |  |
| CESC |  |  | + |
| CHOL | + | + |  |
| COAD |  |  | + |
| DLBC |  |  | + |
| ESCA |  | + |  |
| GBM | + | + |  |
| HNSC | + | + |  |
| KIRC | + |  | + |
| LIHC | + |  | + |
| LUAD |  | + | + |
| LUSC |  | + |  |
| OV |  | + |  |
| PAAD | - |  |  |
| PRAD |  |  | + |
| SARC |  | + |  |
| SKCM |  | + | + |
| STAD | + | + |  |
| THYM | - |  |  |
| UCEC |  | + | + |
| UCS |  | + |  |

Abbreviations: ICB, immune-checkpoint blockade; ACC, adrenocortical carcinoma; BLCA, bladder urothelial carcinoma; CESC, cervical squamous cell carcinoma and endocervical adenocarcinoma; CHOL, cholangiocarcinoma; COAD, colon adenocarcinoma; DBLC, diffuse large B-cell lymphoma; ESCA, esophageal carcinoma; GBM, glioblastoma multiforme; HNSC, head and neck squamous cell carcinoma; KIRC, kidney renal clear cell carcinoma; LIHC, liver hepatocellular carcinoma; LUAD, lung adenocarcinoma; LUSC, lung squamous cell carcinoma; OV, ovarian serous cystadenocarcinoma; PAAD, pancreatic adenocarcinoma; PRAD, prostate adenocarcinoma; SARC, sarcoma; SKCM, skin cutaneous melanoma; STAD, stomach adenocarcinoma; THYM, thymoma; UCEC, uterine corpus endometrial carcinoma; UCS, uterine carcinosarcoma.
