# Supplementary material for: Multi-Omics Analysis of the Anti-tumor Synergistic Mechanism and Potential Application of Immune Checkpoint Blockade Combined With Lenvatinib
Source: Front Cell Dev Biol. 2021 Sep 9;9:730240. doi: 10.3389/fcell.2021.730240 (PMC8458708; doi:10.3389/fcell.2021.730240)
Supplement: Supplementary file 8 [file Table_3.DOCX]

**Supplementary Table 3. Summary of targets for tyrosine kinase inhibitors**

| Drug | Treatment Target | Indication |
| --- | --- | --- |
|  | Multiple targets |  |
| Lenvatinib | VEGFR-1, VEGFR-2, VEGFR-3, PDGFR-α, PDGFR-β, c-KIT, RET, FGFR-1, FGFR-2, FGFR-3, FGFR-4 | RR-DTC, RCC, HCC |
| Sorafenib | VEGFR-1, VEGFR-2, VEGFR-3, PDGFR-β, c-KIT, FLT3, Raf-1, B-Raf, RET | HCC, RCC, DTC |
| Regorafenib | VEGFR-1, VEGFR-2, VEGFR-3, PDGFR-β, c-KIT, TIE-2, FGFR-1, FGFR-2, RET, RAF-1, BRAF, p38, CSF-1R | HCC, Colorectal cancer, GIST |
| Dovitinib | VEGFR-1, VEGFR-2, VEGFR-3, PDGFR-β, c-KIT, FGFR-1, FGFR-3 | HCC |
| Sunitinib | VEGFR-1, VEGFR-2, VEGFR-3, PDGFR-α, PDGFR-β, c-KIT, FLT-3, RET, CSF-1R | RCC, GIST |
| Pazopanib | VEGFR-1, VEGFR-2, VEGFR-3, PDGFR-α, PDGFR-β, c-KIT, FGFR | RCC, Soft tissue sarcoma |
| Vandetanib | VEGFR-2, VEGFR-3, EGFR, RET | Medullary thyroid carcinoma |
| Cabozantinib | VEGFR-1, VEGFR-2, VEGFR-3, ROS1, RET, AXL, NTRK, KIT, MET | DTC, NSCLC, HCC |
| Anlotinib | VEGFR-1, VEGFR-2, VEGFR-3, PDGFR-α, PDGFR-β, c-KIT, FGFR-1, FGFR-2, FGFR-3, FGFR-4 | NSCLC |
| Axitinib | VEGFR-1, VEGFR-2, VEGFR-3, c-KIT | RCC |
| Imatinib | BCR-Abl, c-KIT, PDGFR, v-ABL | Chronic Myelogenous Leukemia, GIST |
| Tivozanib | VEGFR-1, VEGFR-2, VEGFR-3, PDGFR-β, c-KIT | RCC |
| Apatinib | VEGFR-2, c-KIT, RET, c-Src | GC |
| Fruquintinib | VEGFR-1, VEGFR-2, VEGFR-3 | Colorectal cancer |
| Cediranib | VEGFR-1, VEGFR-2, VEGFR-3, c-KIT, FLT-3, PDGFR-α | Ovarian carcinoma |
| Aflibercept | VEGF-A, VEGF-B, PIGF | Colorectal cancer |
| Nintedanib | VEGFR-1, VEGFR-2, VEGFR-3, FGFR-1, FGFR-2, FGFR-3, PDGFR-α, PDGFR-β, Flt-3, Lck, Lyn, Src | NSCLC |
| Pelitinib | EGFR, HER2 | NSCLC, Colon carcinoma |
| Afatinib | EGFR, HER2 | NSCLC, BC |
| Dacomitinib | EGFR, HER2, HER4 | NSCLC |
| Lapatinib | EGFR, HER2 | BC |
| Canertinib | EGFR, HER2, HER3, HER4 | BC |
| Neratinib | EGFR, HER2, HER4 | BC |
| Everolimus | mTOR, HIF1, HIF2, VEGF | RCC |
|  | Single target |  |
| Gefitinib | EGFR | NSCLC |
| Erlotinib | EGFR | NSCLC |
| Icotinib | EGFR | NSCLC |
| Osimertinib | EGFR | NSCLC |
| Bevacizumab | VEGF-A | NSCLC, GC, Colorectal cancer |
| Ramucirumab | VEGFR-2 | GC, Colon carcinoma |
| Cetuximab | EGFR | Colorectal cancer, HNSC |
| Olaratumab | PDGFR-α | Soft tissue sarcoma |

Abbreviations: BC, breast carcinoma; DTC, differentiated thyroid cancer; GC, gastric cancer; GIST, gastrointestinal stromal tumor; HNSC, head and neck squamous cell carcinoma; NSCLC, non-small-cell lung cancer; RCC, renal cell carcinoma; RR-DTC, radioiodine-refractory differentiated thyroid carcinoma.
