# Supplementary material for: Multi-Omics Analysis of the Anti-tumor Synergistic Mechanism and Potential Application of Immune Checkpoint Blockade Combined With Lenvatinib
Source: Front Cell Dev Biol. 2021 Sep 9;9:730240. doi: 10.3389/fcell.2021.730240 (PMC8458708; doi:10.3389/fcell.2021.730240)
Supplement: Supplementary file 9 [file Table_4.DOCX]

**Supplementary Table 4. Comparison of IC50 for multiple tyrosine kinase inhibitors**

| Target | Lenvatinib | Sorafenib | Regorafenib | Anlotinib | Sunitinib | Cabozantinib | Apatinib | Bevacizumab |
| --- | --- | --- | --- | --- | --- | --- | --- | --- |
| VEGFR-1 | 4.7 | 21 | 13 | 26.9 | 71.5 | 5294 | — | 0.15 |
| VEGFR-2 | 3 | 21 | 4.2 | 0.2 | 4 | 0.035 | 1 | 0.15 |
| VEGFR-3 | 2.3 | 16 | 46 | 0.7 | 15.7 | — | — | 0.15 |
| FGFR-1 | 61 | 340 | — | — | — | — | — | — |
| FGFR-2 | 27 | 150 | — | — | — | — | — | — |
| FGFR-3 | 52 | 340 | — | — | — | — | — | — |
| FGFR-4 | 43 | 3400 | — | — | — | — | — | — |
| PDGFR-α | 29 | 1.6 | — | — | — | — | — | — |
| PDGFR-β | 160 | 27 | 22 | 115 | 7.7 | 234 | — | — |
| RET | 6.4 | 15 | 1.5 | — | — | 4 | 13 | — |
| c-KIT | 85 | 140 | 7 | 14.8 | 11 | 4.6 | 429 | — |
