# Supplementary material for: Multi-Omics Analysis of the Anti-tumor Synergistic Mechanism and Potential Application of Immune Checkpoint Blockade Combined With Lenvatinib
Source: Front Cell Dev Biol. 2021 Sep 9;9:730240. doi: 10.3389/fcell.2021.730240 (PMC8458708; doi:10.3389/fcell.2021.730240)
Supplement: Supplementary file 10 [file Table_5.DOCX]

**Supplementary Table 5. Summary of the combination mode of different TKIs**

|  | Type-I | Type-II | Type-III | Type-IV | Type-V |
| --- | --- | --- | --- | --- | --- |
| DFG conformation | In | Out | Out | NE | In |
| Binding region | ATP-binding site | ATP-binding site and  neighboring region | Neighboring region | Allosteric site not adjacent to ATP-binding site | ATP-binding site and  neighboring region |
| ATP competitive | Yes | Yes | No | No | Yes |
| Selectivity | low | High | High | High | High |
| Association kinetics | Rapid | Slow | Slow | NE | Rapid |
| Dissociation kinetics | Rapid | Slow | Slow | NE | Relatively slow |
| Drugs | Dasatinib, Gefitinib, Erlotinib, Crizotinib, AZD4547, Crenolanib, Gilteritinib, Avapritinib, Midostaurin | Lapatinib, Nilotinib, Sorafenib, Ponatinib, Pazopanib, Imatinib, Regorafenib, Sunitinib | Trametinib | NE | Lenvatinib |

Abbreviations: DFG, Asp-Phe-Gly.
