# Supplementary material for: Multi-Omics Analysis of the Anti-tumor Synergistic Mechanism and Potential Application of Immune Checkpoint Blockade Combined With Lenvatinib
Source: Front Cell Dev Biol. 2021 Sep 9;9:730240. doi: 10.3389/fcell.2021.730240 (PMC8458708; doi:10.3389/fcell.2021.730240)
Supplement: Supplementary file 11 [file Table_6.DOCX]

**Supplementary Table 6. On-going clinical trials of lenvatinib**

| Study ID | Phase | Lines | Tumor types | Size | Endpoints | Regimens |
| --- | --- | --- | --- | --- | --- | --- |
| NCT04207086 | Ⅱ |  | Advanced melanoma | 20 | ORR | L+K |
| NCT03418922 | ⅠB |  | HCC | 30 | DLTs, ORR | L+O |
| NCT03006926 | Ⅰ |  | HCC | 104 | PFS, OS, ORR, AE | L+K |
| NCT03841201 | Ⅱ | 1L | Multinodular advanced HCC | 50 | PFS, OS, ORR, AE | L+O |
| NCT04267120 | Ⅱ |  | Advanced or metastatic non-ccRCC | 34 | PFS, OS, ORR, AE | L+K |
| NCT04393350 | Ⅱ |  | Advanced non-metastatic kidney cancer | 17 | OS, ORR, AE | L+K |
| NCT02973997 | Ⅱ |  | Progress RR-DTC | 60 | PFS, OS, ORR, AE | L+K |
| NCT04209660 | Ⅱ |  | Recurrent/Metastatic adenoid cystic carcinoma | 64 | PFS, ORR | L+K |
| NCT03884101 | Ⅲ | 1L | Stage III, IV or recurrent Endometrial carcinoma | 720 | PFS, OS, ORR, AE | L+K  Paclitaxel+Carboplatin |
| NCT03713593 | Ⅲ | 1L | Advanced HCC | 750 | PFS, OS, ORR, AE | L+K  L+Placebo |
| NCT03820986 | Ⅲ | 1L | Advance melanoma | 660 | PFS, OS, ORR, AE | L+K  K+Placebo |
| NCT03776136 | Ⅱ |  | Advanced melanoma | 100 | PFS, OS, ORR, AE | L+K |
| NCT03797326 | Ⅱ |  | TNBC, OV, GC, CRC, GBM, BTC | 180 | PFS, OS, ORR, AE | L+K |
| NCT03829332 | Ⅲ |  | Treatment-navie NSCLC | 620 | PFS, OS, ORR, AE | L+K  K+Placebo |
| NCT03976375 | Ⅲ |  | Metastatic NSCLC | 405 | PFS, OS, ORR, AE | L+K  Docetaxel/L Monotheray |
| NCT04199104 | Ⅲ | 1L | Recurrent/Metastatic HNSC | 500 | PFS, OS, ORR, AE | L+K  K+Placebo |
| NCT03898180 | Ⅲ | 1L | Locally advanced or metastatic urothelial carcinoma | 694 | PFS, OS, ORR, AE | L+K  K+Placebo |
| NCT04246177 | Ⅲ |  | TACE, incurable /non-metastatic HCC | 950 | PFS, OS, ORR, AE | L+K+TACE |
| NCT03609359 | Ⅱ |  | Advanced GC | 29 | PFS, OS, ORR, AE | L+K |
| NCT03895970 | Ⅱ |  | Advanced hepatobiliary tumors, BTC | 50 | PFS, OS, ORR | L+K |
| NCT04368078 | Ⅱ |  | Advanced HCC | 76 | PFS, OS, ORR, AE | L+Toripalimab |
| NCT04287829 | Ⅱ | 2L/3L | Malignant pleural mesothelioma | 36 | PFS, ORR, AE | L+K |
| NCT02501096 | ⅠB/Ⅱ |  | NSCLC, RCC, Endometrial carcinoma, Urothelial carcinoma, HNSC, melanoma | 357 | PFS, OS, ORR, TEAE | L+K |
| NCT04401800 | Ⅱ |  | Advanced or metastatic HCC | 66 | PFS, ORR, AE | L+Tislelizumab |
| NCT04042805 | Ⅱ |  | Locally advanced HCC | 56 | OS, ORR, AE | L+Sintilimab |
| NCT04211168 | Ⅱ | 2L | Advanced BTC | 44 | PFS, OS, ORR, AE | L+Toripalimab |
| NCT04361331 | Ⅱ |  | Advanced intrahepatic BTC | 60 | PFS, OS, ORR, AE | L+Toripalimab |
|  |  |  |  |  |  | L+Gemox |

Abbreviations: AE, adverse drug event; BC, breast carcinoma; BTC, biliary tract carcinoma; CRC, colorectal cancer; DLTs, dose-limiting toxicitys; DTC, differentiated thyroid cancer; GC, gastric cancer; GBM, glioblastoma multiforme; GIST, gastrointestinal stromal tumor; HNSC, head and neck squamous cell carcinoma; K, pembrolizumab; L, lenvatinib; NSCLC, non-small-cell lung cancer; O, nivolumab; ORR, objective response rate; OS, overall survival; OV, ovarian serous cystadenocarcinoma; PFS, progression free survival; RCC, renal cell carcinoma; RR-DTC, radioiodine-refractory differentiated thyroid carcinoma; TACE, transarterial chemoembolization; TEAE, treatment emergent adverse events; TNBC, triple-negative breast carcinoma.
