# Supplementary material for: Multi-Omics Analysis of the Anti-tumor Synergistic Mechanism and Potential Application of Immune Checkpoint Blockade Combined With Lenvatinib
Source: Front Cell Dev Biol. 2021 Sep 9;9:730240. doi: 10.3389/fcell.2021.730240 (PMC8458708; doi:10.3389/fcell.2021.730240)
Supplement: Supplementary file 12 [file Table_7.DOCX]

**Supplementary Table 7. Potential dual-targets for cancer treatment**

| Tumor | High expression of ICB targets | High expression of lenvatinib targets | Correlation of targets | Driver mutation of lenvatinib targets | Total mutation load of the targets |
| --- | --- | --- | --- | --- | --- |
| LIHC | PD-1 | FGFR3/FGFR4 | + | + | 28 |
| GBM | PD-L1 | VEGFR2 | + | + | 46 |
| STAD | PD-L1 | FGFR2 | - | + | 64 |
| SKCM | CTLA4 | PDGFRA | + | + | 121 |

Abbreviations: CTLA-4, cytotoxic T-lymphocyte antigen-4; GBM, glioblastoma multiforme; LIHC, liver hepatocellular carcinom; PD-1, programmed cell death 1; PD-L1, programmed cell death ligand 1; SKCM, skin cutaneous melanoma; STAD, stomach adenocarcinoma.
